# Overcoming medical scholasticism in New Spain: experience and indigenous knowledge in Arias de Benavides’ treatment of syphilis

**DOI:** 10.1007/s40656-025-00699-x

**Published:** 2026-03-23

**Authors:** Manuel Méndez Alonzo, Luis Alejandro Villanueva

**Affiliations:** 1 Department of History, University of Campinas, Rua Coralina 100, 13086896 Campinas São Paulo, Brazil; 2 Institute of Cultural History, Research Centre of the Slovenian Academy of Sciences and Arts, Novi Trg, 2, 1000 Ljubljana, Slovenia; 3 Chair for Computational and Theoretical Biology, CCTB, Julius-Maximilians-Universität Würzburg, Klara-Oppenheimer-Weg 32, Campus Hubland Nord, 97074 Würzburg, Germany; 4 Konrad Lorenz Institute for Evolution and Cognition Research, KLI, Martinstraße 12, 3400 Klosterneuburg, Austria

**Keywords:** Non-Western medical knowledge, Pedro Arias de Benavides, American *materia medica*, Syphilis in the New World, Maguey

## Abstract

This article examines the impact of non-Western medical knowledge on European medicine during the Early Modern period, with a specific focus on the medical work of Spanish surgeon Pedro Arias de Benavides in New Spain. The paper discusses Benavides' innovative approach to treating syphilis in the New World, which challenged the inefficiencies of European scholastic medicine when dealing with diseases prevalent in the Americas. It shows that Benavides´scientific contribution stems from the assimilation of indigenous healing practices and American *materia medica* into his medical background, often questioning established classical medical theories.

## Introduction

There is a growing recognition that the history of science should not be seen merely as an account of the development and worldwide dissemination of Western knowledge. What was once portrayed as a linear process of Western expansion—marked, among other things, by the imposition of European knowledge on other parts of the world—is now approached with greater nuance. Rather than a unidirectional transfer from dominant centers to “passive” peripheries, these historical processes often gave rise to intricate exchanges among diverse knowledge traditions, each shaped by its own cultural and historical specificity (Saldaña, 2001). Latin America is particularly significant, as its historical and distinctive mixture of cultural traditions—including European, Indigenous, and African influences—has given rise to a rich confluence of knowledge systems (Freire Junior & Paty, 2024). Approaching this phenomenon through the lens of the history of science has revealed not only the existence of multiple ways of producing scientific knowledge, but also that efforts to impose modern Western science have never resulted in homogeneous outcomes (Saldaña, 2001). The history and philosophy of medicine offer valuable insights for analyzing these complex dynamics. Throughout the processes of European imperial expansion, Western medicine faced critical scrutiny—prompting not only a reconfiguration of its therapeutic practices but also a profound challenge to its philosophical foundations. However, the examination of these processes remains a fledgling area of research. The study of the work carried out by physicians in the Spanish American colonies can provide significant contributions to furthering this endeavour. To advance in this direction, this paper discusses the theoretical and medical relevance of the only published work by the Spanish surgeon Pedro Arias de Benavides: *Secretos de Chirurgia, especial de las enfermedades de Morbo gálico y Lamparones y Mirrachia, y así mismo como se curan los indios las llagas y heridas y otras pasiones en Las Indias, muy útil y provechoso para España y otros muchos Secretos de Chirurgia hasta ahora no escritos*,^1^ hereinafter *Secretos*, published in Valladolid, Spain, in 1567.^2^ This book is one of the earliest works written by medical personnel interested in showcasing the therapeutic techniques and *materia medica* discovered in the New World.^3^

Until now, only a limited number of studies have been conducted on the life and specific aspects of the work of Pedro Arias de Benavides. This includes the article “La materia médica americana en la obra de Arias de Benavides" by Valverde et al., published in 1986, that provides a general account of the plants mentioned by the surgeon. On the other hand, the monograph entitled *La experiencia americana y la terapéutica en los Secretos de Chirurgia *(1567)* de Pedro Arias de Benavides* by José Luis Fresquet Febrer, published in 1993 by the University of Valencia, provides a general overview of *Secretos* and the historical context in which Benavides´work appeared. More recently, the paper "First Published Record of a Neurosurgical Procedure on the North American Continent, Mexico City, by Pedro Arias de Benavides, 1561: Secretos de Chirurgia, Valladolid, Spain, 1567” by De León et al. (2000), analyzes Benavides's contributions as a neurosurgeon. Also in the year 2020, Yarí Pérez Marín published a book with the title *Marvels of Medicine*: *Literature and Scientific Enquiry in Early Colonial Spanish America*. The first chapter of the book is dedicated to Arias de Benavides, presenting his work as a collection of medical secrets and a defense of the achievements of the surgical profession. In 2023, Bau published the paper “Lecturas de la alteridad. Cuerpos e identidades en el discurso de un cirujano en América, Pedro Arias de Benavides”. This article analyzes Benavides' work as an example of how the Western medical perspective established a relationship between the bodies of indigenous peoples of the Americas, their local environments, and the illnesses found in the New World. Undoubtedly, these studies have significantly contributed to our knowledge of certain aspects of Benavides' life, work, and historical context. However, literature on this topic remains scarce, and a deeper analysis is necessary to fully appreciate the significance of Benavides’ critiques and contributions to European academic medicine. This paper addresses this issue by examining how effective Benavides’s medical approach was in treating diseases in the Americas—where European academic medicine often failed—and, more importantly, how his empirical use of Indigenous healing practices helped challenge the rigid theoretical principles of scholastic medicine. Special attention will be placed on the knowledge Benavides acquired about the therapeutic properties of plants native to the Indies, with particular emphasis on his use of Maguey (agave) in the treatment and cure of syphilis.^4^

To this end, the paper is organized as follows. Section 2 provides an overview of how humoral theory shaped the principles of modern medicine and the ways in which these principles contributed to the professionalization of medicine in certain regions of Europe and Spain. It then highlights the most important similarities and differences between these medical systems and the practices of the Indigenous peoples of the New World during the sixteenth century. Section 3, reviews the medical context in which Pedro Arias de Benavides developed his career in New Spain, as well as the intellectual and practical motivations that influenced his work. Subsequently, Sect. 4, outlines how the spread of syphilis in the Americas prompted Benavides to develop his novel approach to medicine. Afterwards, Sect. 5, presents Benavides' perspective on the primary causes and symptoms of syphilis, a diagnosis that motivated the need for new medical treatments. Section 6, discusses how Benavides' integration of Spanish and indigenous medicine led to a significant improvement in the diagnosis and treatment of syphilis in the Americas. Section 7 examines Benavides’use of local anecdotes—not only to advocate for the higher effectiveness of Indigenous healers, but also to highlight how the shortcomings of European medicine fostered deep mistrust among the inhabitants of the Indies. Finally, in Sect. 8, we present our conclusions.

## Classical legacy, medical professionalization in Europe, and indigenous healing knowledge: parallels and differences

The roots of medical theory in early modern Europe lie in classical antiquity, particularly in the works of Galen and Hippocrates, whose influence shaped the understanding of health as a state of equilibrium between the four bodily humors: blood, phlegm, black bile, and yellow bile. Disease was seen as an imbalance between principles within the body (such as cold, hot, dry, and humidity). Imbalance was produced by external factors, such as food, climate, and personal lifestyle. Healing was conceived as the process of recovering the body's balance (Johnston, 2006, p. 89). Thus, physicians were expected to treat diseases and prescribe instructions on how to maintain a balanced way of life (Cavallo, 2011, p. 197). In the Middle Ages, the emergence of universities opened the door for laypeople to study medicine. Scholastic medicine, drawing on the classical figures, emphasized logical analysis and direct observation in understanding the human body. Over time, medicine and surgery came to be seen as separate professions. Physicians were expected to be literate, trained in Latin, and well-versed in classical medical texts. In contrast, surgeons were simply regarded as practitioners who performed manual labor. This distinction corresponded to differing entry requirements for each profession. For instance, starting from the fourteenth century, a university education became a prerequisite for joining the physicians' guild (Siraisi, 1990, p. 18). This led to the creation of a hierarchical structure within the discipline: "University graduates in medicine occupied the highest position, followed by other skilled medical practitioners, then by skilled surgeons, and finally by barber-surgeons and various other practitioners, including herbalists and apothecaries." (Siraisi, 1990, p. 18).

In the years that followed, certain intellectual contexts fostered distinct developments of medical training. For instance, by the mid-sixteenth century, while much of the instruction in medicine at the University of Padua was guided by the study of classical texts by Galen, Hippocrates, and Avicenna, other lectures also drew on texts focused on the practical treatment of aiments—such as Antonio Fracanzani’s treatise on fevers, Alvise Bellocato’s on apoplexy, and Vettore Trincavelli’s on illnesses caused by worms and on arthritis (Stolberg, 2014, p. 638). In 1537, Andreas Vesalius was appointed to the chair of surgery, with the responsibility for teaching anatomy. His treatise *De humani corporis fabrica libri septem* (1543) is widely regarded as the foundation of modern anatomy. Vesalius’s work identified a series of errors in Galen’s writings, based on direct observation and experience acquired through the dissection of human cadavers. In his lectures, he taught students to prioritize empirical evidence over theoretical speculation (FitzGibbon, 1951). It is important to remark that Vesalius’ treatise soon became a principal reference for anatomical studies and a key scholarly resource for Benavides (Lazzerini, 1994, pp. 195–196).

Moreover, Stolberg (2014), based on a careful study of lecture notes from various students at the University of Padua, found that professors used to take their students on visits to patients’ homes and hospitals. These visits allowed students to physically examine the patients, assess their conditions and bodily symptoms, and monitor the progression of the illness through repeated visits. In sum, a fundamental principle in this medical training was that the “mind” should not be separated from the “hand” (Stolberg, 2014, p. 649). In this context, medicine was understood not merely as a body of universal theories, but as a discipline rooted in their application to individual cases—something that could only be tested through experience. These developments marked significant efforts to transform the professionalization of medicine. Nevertheless, disciplinary boundaries remained. In cities such as Padua and Bologna, “educated” surgeons were typically affiliated with the College of Physicians, while unlicensed or non-graduate surgeons were associated with Barbers' Guilds. Likewise, in many parts of Europe—particularly in Venice and several cities in Northern Europe—a clear distinction persisted among the Colleges of Physicians, the Colleges of Surgeons, and the Guilds of Barber-Surgeons (Savoia, 2019, p. 29).

In sixteenth-century Spain, the medical profession was also influenced by both medieval and classical Greco-Roman medical thinking. Spanish physicians began to show a reformist spirit in response to these influences, while avoiding any conflict with Aristotelian philosophy (Domingues, 1997). In light of this, physicians sought to establish connections between scholastic philosophy and recent discoveries in the natural sciences to restructure their discipline (Domingues, 1998, p.300). Nonetheless, canonical Arab translations came under scrutiny and were regarded as unreliable. Thus, unlike the medical context in Padua described above, Spanish physicians concentrated their efforts on translating the works of Galen, Hippocrates, and Dioscorides, rendering the practice of medicine more reliant on the exegesis of classical texts than on experience (Fresquet, 1995, p. 75).

Upon the arrival of the Spaniards in the New World, physicians discovered that the local cultures had well-structured and complex medical systems. This situation presented a unique opportunity to expand the Spanish *materia medica* and therapeutic knowledge.^5^ However, not all medical practices in the Indies were entirely new. Spanish and Mesoamerican medical traditions share many common aspects, including a view of health as a balance of the body^6^ that could be affected by external influences and immoral behavior (Estrella, 1992, p. 13). This is evident in the notion of health (*pacayeliztli*) as a delicate equilibrium between the spiritual and natural realms—a widespread idea across Mesoamerican cultures. In this worldview, disease (*cocoliztli*) was seen as determined by the individual's moral conduct. Thus, Mexica people believed that when a patient succumbed to illness, the healer was not held responsible, but rather it was attributed to the patient's lack of faith, wickedness, or curses (Jaen & Murillo, 2005, p. 889). The idea that good health was maintained through ethical conduct, while disease functioned as a punishment for violating moral and religious norms, was deeply rooted among indigenous populations (Estrella, 1992, p. 13). In addition to moral and spiritual causes, witchcraft was believed to account for many illnesses, with plants and animals also seen as agents of disease.

Furthermore, much like the European humoral theory, indigenous practitioners also believed that diseases arose from a duality of cold and hot elements (López Austin, 1993, pp. 17–18). Hence, dietary factors were not only seen as generators of diseases but also as playing an important role in their treatment (López Austin, 1993, p. 21–22). Additionally, Mesoamerican cultures conceived diseases (*malos aires*) as being caused by external entities (Basset, 2015, p. 28) capable of inflicting retribution—targeting individuals or groups, and as such, treated with both reverence and fear (Estrella, 1992, pp. 18–19). Thus, in the Mexica society, illnesses and epidemics were associated with *Tezcatlipoca*, while *Centeotl* was revered as the guardian of medicinal herbs, worshipped by surgeons, midwives, and healers (Estrella, 1992, p. 21–22). Based on this view, each part of the body was associated with particular gods responsible for its healing. For this reason, medical practitioners were expected to possess theological specialization, as treatment often incorporated the invocation of deities and the use of protective amulets.

It is important to remark that indigenous healing traditions also gave considerable weight to knowledge based on empirical experience. Medicine was the domain of skilled men and women, mainly recognized for their religious wisdom and empirical knowledge. Thus, unlike the strict division of roles characteristic of medical institutionalization in Spain, Mesoamerican healers were expected to possess a close familiarity with magical rituals, as well as specialized knowledge of herbal properties and therapeutic techniques. This more integrated form of medical practice found in Mesoamerican cultures, although conceptually aligned with humoral theory and thus with the theoretical foundations of Spanish medicine, was—through its openness to technical procedures—more closely related to the type of medicine emerging in Padua. The arrival of the Spaniards in the Americas—and, among other factors, the shortage of medical personnel to address local needs—made possible the emergence of “spaces exchange” and communication between the Mesoamerican and Spanish medical traditions (Pardo-Tomás, 2013, p. 22). The following section outlines the intellectual context and empirical motivations that shaped Benavides’s professional trajectory and nurtured his openness to incorporating the practical approach of Indigenous healing into his medical work.

## Benavides’s professional trajectory in the medical context of New Spain

Before the establishment of the first medical chair in Mexico, the interaction between indigenous healers and trained Spanish practitioners was encouraged by the urgent need in New Spain to train medical personnel and to explore the botanical and pharmaceutical resources of the Americas as alternatives to traditional remedies mainly imported from the East (Boumediene & Pugliano, 2019, p. 25). In this context, the Universidad Real y Pontificia de México was founded in 1553.^7^ During its first years, it followed the curriculum model of the University of Salamanca and, for a period of 25 years, gave greater emphasis to the teaching of law and theology, while relegating natural philosophy and medicine (González González, 1992, p. 155). It was not until 1578 that the first academic chair of medicine in New Spain was established at the University of Mexico. In that year, Juan de la Fuente became the first incumbent of the chair of medicine (González González, 1992, p. 158). He renovated the curricula of the university and included five new subjects: the origins of medicine*, metodo medendi*, anatomy and surgery, astrology, and mathematics (Rodríguez, 1992, p. 181–185). In medicine, humoralism was the main theoretical approach.^8^ In general, the Spanish colonies followed the model that had been established in Spain, which was made up of different specialties: academic physicians (responsible for medical treatment) as well as barbers, midwives, and other healthcare assistants, who performed practical duties (Fresquet, 1995, p. 24). Surgery was considered an empirical practical trade, performed under the supervision of a physician with academic education (Rodríguez-Sala et al., 2006, p. 23). In other words, surgeons were always expected to operate under the scrutiny of physicians. Thus, while physicians were responsible for making diagnoses, surgeons handled the practical tasks (Rodríguez-Sala et al., 2006, p. 25–26). Nevertheless, it is important to highlight that, whenever possible, surgeons often sought to demonstrate the medical limitations of the classic texts used by academic medicine by weighing the theses of the authorities against clinical evidence. In essence, surgeons argued that classical medical authorities should be subordinated to personal observation and experience (López Piñero, 1992, p. 63). In this context, where a climate of criticism toward the classical authorities was developing, the contribution of Benavides’s work stands out. This contribution, in turn, would not have been possible without the multicultural conditions of New Spain, which allowed academic medicine to coexist with the knowledge of the indigenous peoples of the Americas and the medical practices of the growing community of African slaves (Hernández Saenz & Foster, 2001, p. 23). Benavides’ endorsement of the therapeutic practices of Indigenous peoples—particularly the Nahuas of Mexico—illustrates, as McDonough (2024, chap. 2) rightly observes, the enduring influence of these medical traditions on post-conquest healthcare practices.

Pedro Arias de Benavides was born around 1521 in El Toro, a small town in the Spanish Municipality of Zamora. His departure for the New World can be tentatively dated between 1545 and 1550. This journey encompassed the Canary Islands, Santo Domingo, Honduras, Guatemala, and Mexico. His arrival in Guatemala can be dated in 1550. He worked there as a surgeon for four years. He then arrived in Mexico around 1554 (Somolinos-Palencia, 1992, p. 4–5). During his stay in Mexico City, he spent several years at the *Hospital del Amor Dios* or *Hospital de Bubas* (Okholm Skaarup, 2015, p. 55). It is unclear when Benavides returned to Spain and to El Toro, as he did not mention a specific date. However, it is possible that this occurred between 1562 and 1564 (Fresquet, 1993, p. 55). It is known that Benavides was well versed in the medical theories developed by Galen, Dioscorides, and Hippocrates (Fresquet, 1993, p. 55). He subscribed to the humoral theory of health. There is limited information on the professional life of Arias de Benavides in Spain. However, evidence suggests that despite possessing a strong understanding of humanist textbooks and accepting the authority of classical authors such as Galen, Dioscorides and Hippocrates, he prioritized the importance of experience over pure theory.^9^ This aligns with the new interpretation of Galen's philosophy during the Renaissance in Spain,^10^ which helps explain Benavides willingness to incorporate American indigenous healing techniques within medical general knowledge. The scientific openness of Benavides can also be partly attributed to his desire to uphold the parity of the surgeons' guild vis-à-vis the purported supremacy of scholastic medical thought, which resonates with the value he placed on the work of surgeons such as Guy de Chauliac, Giovanni Vigo, Rogerio de Salerno, Ruy Díaz de la Isla, Guglielmo de Saliceto, and Andrea Vesalius (Fresquet, 1993, p. 29). These intellectual, institutional, and practical motivations led Benavides to express in *Secretos* his determination to demonstrate the relevance of appropriating the medical resources of Mesoamerican cultures for addressing the health challenges faced by Spain and its colonies.

To conclude this section, it is important to note that, although *Secretos* did not have the same impact as the works of Nicolas Monardes^11^ or Francisco Hernández,^12^ it is regarded as one of the earliest accounts of the medical therapies and remedies of the Indies—particularly those of New Spain and the Antilles. Its publication in Valladolid further suggests that it garnered attention from contemporary medical circles. In the late sixteenth century, the University of Valladolid emerged as a key center not only for disseminating the works of Galen and Hippocrates but also for advancing anatomical studies, led by prominent figures such as Alfonso Rodríguez de Guevara. In 1551, Bernardino Montaña de Monserrate, surgeon to Emperor Charles V, also published *Libro de la Anathomía del hombre*, the first anatomy book written in Spanish. The city was also distinguished by its strong surgical tradition and well-stocked medical libraries, including those of Pedro Martínez and professors Francisco Martínez Polo and Octavio Sanz de Soria, which held hundreds to over a thousand medical volumes (Vaquero Puerta, 2022, pp. 71-73). It is very likely that this intellectual environment received with great interest the novel medical approach proposed in *Secretos.*

## The spread of syphilis in the Indies and the limitations of classical medicine to treat it

A major outbreak during the Italian Wars was the syphilis epidemic that occurred in Europe in 1495 during the siege of Naples by the French (Tognotti, 2009, p. 100). The rapid spread of the disease presented a significant challenge for European academic medicine. Physicians were able to effectively examine its development in the body, but its causes remained unknown (Tognotti, 2009, p. 100–101). Most physicians and surgeons believed that the infection had a venereal origin. Syphilis, then, was often seen as a divine punishment for sexual promiscuity (Arrizabalaga et al., 1997; Schleiner, 1994, p. 399). It was believed that abstaining from engaging with prostitutes was the safest way to prevent it. Medical prescriptions included moral admonitions directed at those perceived as licentious, as well as warnings against illicit sexual encounters (Schleiner, 1994, p. 406). In broad terms, these prescriptions reflect a moral critique of lifestyle, associating the disease with poverty and moral failings (Arrizabalaga et al., 1997, p. 26).

In the sixteenth century, the origins of syphilis were also debated in Spanish colonies.^13^ Some believed it had a pre-Columbian origin, while others believed it originated in Europe (Nun & Qian, 2010, p. 166–167). By 1560, due to the work of pharmacists and apothecaries in Italy and Aragon, more than 400 substitute remedies were available to medical personnel for treating this disease (Boumediene & Pugliano, 2019, p. 31). In 1570, Francisco Hernández traveled to the Americas to catalogue medicinal plants that could replace scarce remedies in Europe (Boumediene & Pugliano, 2019, p. 31–32). However, Benavides believed that the benefits of the American medicinal plants should not be seen merely as a substitute for the European pharmacy. In contrast, he argued that practical knowledge of New World medicinal plants was therapeutically superior to Classical medicine. Rejecting academic orthodoxy, he promoted indigenous remedies—including a possible cure for syphilis.

Despite his practical approach, Benavides did not attempt to develop a completely innovative conceptual interpretation of syphilis. He followed the interpretations of Giovanni de Vigo and Rui de la Isla on the American origin of syphilis (Fresquet, 1993, p. 119–120) and expressed his affinity with Vesalius. Likewise, he recognized his indebtedness to Greco-Roman and Medieval authorities.^14^ However, he believed that the ancients, due to the limitations of their era, could not have possibly possessed knowledge about all the properties of many substances around the world (Fresquet, 2002, p. 258). In other words, he pointed out that while the wisdom of the classics provided guidance and a methodology for applying medical principles, the true effectiveness of a treatment should be determined by practical experience and not solely on the opinion of the medical authorities (López Piñero, 1992, p. 73). As noted, Benavides aimed to preserve the link with the theoretical foundations of Western medicine while highlighting the exigencies of practical experience (Fresquet, 2002, p. 258). He believed that the therapeutic knowledge of American indigenous peoples would offer valuable support for medical progress. With this in mind, he explored the therapeutic resources of American indigenous people. He compared his quest for American resources to voyages of exploration, as an appeal to the search for new forms of healing outside the conventions of the classics (Pérez Marín, 2020, p. 36). In *Secretos,* the author analyzed the characteristics of the social and natural environment of the New World that he believed were the main causes of the spread of syphilis in the Americas.^15^ The next section is an overview of Benavides' diagnosis of the problem and his preliminary measures for its treatment.

## Reported causes and symptoms of syphilitic disease discussed by Benavides

Benavides’ initial reports on syphilis were drawn from his stay in Santo Domingo. The author described the island as having bubonic plague and noted that it was a place where Spaniards coexisted with Africans and Native Americans. The author stated that in Santo Domingo, Spaniards consumed the food of other groups and in some cases were breast-fed by black women to the point of changing their skin color.^16^ In his reflections on the climate and social life of Santo Domingo, Benavides also criticized the islands’ racially mixed society.^17^ He believed that the virulence of syphilis was caused by the constant contact that Spaniards had from an early age with people of different ethnic backgrounds.

All individuals born in this region do not possess a flawless white complexion but rather one that is tanned (*amulatada*)...the individuals who arrive here are at a significant risk. Although the climate is as hot as I have described, almost every food item contains a substantial amount of chili or peppers from the Indians, as well as other dishes with similar ingredients. As a result, there is a substantial prevalence of recurring sores in both Black individuals and Spaniards (Benavides, fol. 10).

Thus, the author considered that the primary source of syphilis contagion was sexual contact with prostitutes, referred to as "unclean" women. This perspective was widely shared among Spanish physicians and moralists of that period. They believed that the rapid proliferation of sexually transmitted diseases and the depopulation of Native American territories could be attributed to the dissolute lifestyles of the Indies' inhabitants (Méndez-Alonzo & Villanueva, 2022, p. 496–497). Although several diseases, including syphilis, were perceived as being caused by a corruption resulting from specific climatic conditions,^18^ their heightened virulence was regarded as a consequence of immoral living.^19^ Benavides believed that “immoral” factors contributed to the spread of the syphilis epidemic, but he pointed out that the most significant issue was the climate of Hispaniola, characterized as extremely hot and humid. Following the humoral theory, the author claimed that this climate was conducive to the development of buboes, and therefore, recommended that individuals with syphilis relocate from hot to cold environments. A relatively cooler climate, he argued, would be more favorable for expelling the malignant humors responsible for syphilis, as the body would be stronger, more resilient, and the spirits more composed.

On the other hand, he attributed the development of syphilis's buboes to an excess of melancholic moods, which Benavides deemed the "most obstinate and earthy." He pointed out that in the case of individuals with melancholic temperaments, the treatment of syphilis proves to be notably challenging.^20^ Conversely, Benavides believed that in cold climates, the spirits and vitality become stronger, and internal heat is better preserved.^21^ He thought that the extreme heat of some regions of the Indies consumed the humidity of the body, revitalizing older people.

Note that despite the challenges posed by the little knowledge available of this disease, Benavides identified several patterns of syphilis: (a) that it is more virulent in young people than in the enderly, primarily because, he believes, young people have blood that is more easily overheated, increasing its susceptibility to corruption; (b) that additional symptoms of syphilis include genital discomfort, joint pain, loss of eyelashes and eyebrows, the presence of sores, impaired digestion, feverless headaches, lumps on the head, and a general feeling of lethargy.^22^ Benavides attributed the physical pains of syphilis to an excess of humors in the blood. He argued that women recovered from syphilis faster than men because they expelled them during menstruation.^23^ Furthermore, he identified three variants of syphilis. The first is caused by an abundance of phlegmatic (non-melancholic) humor. The second is produced, and mainly seen in European men, *per paulatim congestionen*.^24^ The third is due to the formation of protuberances on the skull caused by thick buboes settling on the head.

After this analysis, Benavides recommended frequent bleeding, purgatives, and sour pills and suggested draining buboes, especially those on the skull, to prevent them from corrupting and corroding. He also proposed patches filled with ammonia and melilot^25^ (*melilotus officinalis*) to dissolve the protuberances on the skull. One of his remedies was to drink stews of sarsaparilla twice a day, once in the morning and once at night. He also advised patients to abstain from foods that generate melancholic humors, such as salty dishes, legumes, beans, beef, and all things made of milk.^26^ Among other remedies, he recommended replenishing the blood with borage syrup (*Borrago officinalis*) and common fumitory or earth smoke plant (*Fumaria officinalis*).^27^ For the diet, he suggested a solution of Indian electuary, honey, sugar, and wine. He also recommended lamb cooked in broth to cleanse the liver through urination, which should be monitored for color.^28^ In the case of buboes, if they do not contain a corrosive substance, Benavides proposed draining them. In cases where sores exist, he spoke favorably of bloodletting.^29^ In *Secretos*, Benavides emphasized the success of his treatments in restoring the health of patients. The next section overviews Benavides' rationale for the use of the empirical knowledge of indigenous healers on local products to improve the treatment of this medical problem.

## Benavides’s evaluation of native American healing techniques: maguey and the treatment of Syphilis Buboes

In the initial chapters of *Secretos*, in which Benavides recounts his journey to Santo Domingo, he focuses not only on the syphilis epidemic among both the white and black populations, but also on the techniques used by the latter group to treat and cure the ulcers associated with the disease. The techniques involved the use of ointments made from *berraza* (*apium nodiflorum*) and *cebadilla* (*schoenocaulum officinale*). These ointments were prepared by extracting the juices from these plants and then mixing them with wax and oil.^30^ The bark of this plant is highly caustic and toxic, which is why it was exclusively utilized by Black and American indigenous populations. Individuals who developed buboes also employed a plant known as *hierba hedionda*. To heal syphilis, the Natives set a small fire and, afterwards, placed the *hierba hedionda* over it. Then, the patient was positioned above the hole to inhale the smoke, facilitating the expulsion of harmful humors through sweating and sneezing.^31^

Benavides reported that another plant, widely used by Native Americans in the Caribbean for syphilis treatment, was *Palo de Indias* or *Guayacán* (*Guaiacum officinalis*).The juice was extracted from the *Guayacán* tree and diluted with water. The patient was then given the juice to drink.^32^ In this case, Benavides expressed doubt regarding its effectiveness and refrains from commenting on its therapeutic use.^33^ On the other hand, he also mentioned the treatments he observed in Guatemala for buboes. One remedy involved consuming sarsaparilla (*smilax aspera*) water combined with mercury, without any other food, for a period of three days.^34^ This induced sweating which, in the surgeon's view, expels the syphilitic humors within the same time lapse.^35^ The sarsaparilla was then combined with wine or water for a two-month period. Benavides particularly recommended this technique because it minimized the time the patient must spend in bed.^36^

In addition, Benavides approvingly described two healing techniques of Native Americans in Mexico to cure syphilis. One of them was to purge melancholic humor with fumaria (*Fumaria officinalis*) pills containing *palomina* (*echium plantagineum*). The hard consistency of these pills attracted the humors, as they were melted by the stomach. According to Benavides, Native American healers performed this treatment in conjunction or opposition with the moon. They also purged their patients and made them drink cooked anise, fennel, or sarsaparilla (*smilax aspera*). Another valid method for Benavides was mercury combined with *triaca* (an antidote of 50 plants that includes snake meat), which reduced the poisonous effects of the mineral.^37^

Nevertheless, the technique that most captivated Benavides was the treatment of syphilis using agave leaves, as employed by the indigenous people of central Mexico. He acclaimed the use of maguey (*agave salmiana*) with his peers, asserting that there is no plant in the world with as many virtues and beneficial properties, to the extent that he called it *el mayor tesoro de las Indias* (the greatest treasure of the Indies).^38^ At this juncture, he criticized the doctors of New Spain for their reluctance and dogmatism in experimenting with this plant. He claimed that this reluctance was due to the supposed unknown effects of the plant which, in turn, arose from the lack of academic literature on the subject. However, Benavides's experience with maguey was consistently positive (“*por Dios bendito siempre me fue bien con él”*), advocating and validating its use for medical purposes, despite the absence of references in the classical literature.^39^ Furthermore, Benavides accused physicians of being reluctant to use this plant for medical purposes due to their own self-interest. He pointed out that, by discrediting competitors and their healing techniques, physicians were able to maintain their status and economic earnings:

This maguey, as I have mentioned, has many benefits that I have experienced, yet nobody wanted to explore it because the doctors of that land do not hold it in high regard, due to their interests and gains. When discussing this agave with them, they would tell me... that if I were to ever face an adverse outcome in any treatment, I had nothing in writing to save me from the error, as it is an unknown entity. By God's grace, it has always worked well for me (Benavides, fol. 40).

After explaining the methods of utilizing the plant and the extraction of its juice, which the Indians used for alleviating flu and later for preparing *pulque* (an alcoholic beverage), Benavides underscored the various uses of maguey. Its leaves, or *pencas*, he says, can be employed for roofing houses, making hemp, ropes, and extracting logs. However, according to Benavides, the most crucial property of maguey, known only to the American indigenous healers in Mexico, is its remarkable efficacy in curing syphilis buboes. He elucidated that the Indians cut a lump of maguey, cook it with water for four hours, and then have the patient aspirate the steam to retain the heat. This procedure was repeated for eight days until the patient was healed. Another method reported by Benavides involved burning maguey fragments in a hole and placing the affected person on top of the fire to inhale the smoke. He considered this method more challenging to endure but also more effective, as it cured syphilis in just three days.

They (and I have done so myself) took a lump of the maguey... and put it in a large pot of water... and covered it with a plate and plastered it with dough so that the steam would not escape. There it cooked for three or four hours, and then they took it to where the sick person was and uncovered the pot. A great quantity of steam came out, and the sick person received it. Immediately, he began to sweat, as much as if he were immersed in a river. And then, as he was, they wrapped him up so he sweated. In this way, he was cured of his *bubas* in seven days... and they came out well cured. Other times, they took a [maguey] leaf and made a hole in the ground. They made a lot of embers and put the leaf on top... to roast. They put the Indian on top, so that he would take the heat and sweat with it. Although it was a great effort and hard to endure, it is shorter, because it was done in only three days. In this way, they got over their illness (Benavides, fol. 41-42).

In addition to its therapeutic uses, Benavides mentioned how the maguey was used for house construction and its spines as needles.^40^ Apart from its medicinal use against syphilis, there is evidence that the Nahua peoples of central Mexico considered this plant as an antibacterial remedy, an inflammatory and ritual food (Davidson, & Ortiz, 1983; Vázquez García et al., 2016; Monterrosas-Brisson et al., 2013).^41^ Throughout the mid-sixteenth century, Indigenous health practices gained increasing acceptance. This phenomenon, referred to as *medical mestizaje* by Martínez Hernández (2022, p. 182), describes the gradual incorporation of Native American therapeutic knowledge into colonial medicine. Benavides's appraisal of the therapeutic uses of maguey can be seen as one of the early precedents of this process, as he openly challenged physicians’ orthodoxy and their presumed superiority over other medical traditions. Importantly, as a surgeon, Benavides lacked the theoretical tools to effectively contest the dogmatic principles upheld by university-trained physicians. Most likely, for this reason, Benavides supported his criticism with local anecdotes that expressed the inefficiency of European doctors and the lack of trust in them among the people of New Spain. This issue will be addressed in the next section.

## Benavides’ critique of Spanish medicine in the light of anecdotes from the Indies

Local anecdotes were commonly used by surgeons between the thirteenth and sixteenth centuries. These served multiple purposes, including showcasing the author's successes and criticizing the limitations of other medical practitioners, particularly university-educated physicians (Siraisi, 1990, p. 171–172). The animosity between surgeons and physicians was also due to a “sharp institutional and economic rivalry” (Siraisi, 1990, p. 174). The main purpose of the anecdotes included in *Secretos* was to illustrate the inefficiency of academic medical knowledge by recounting humorous scenarios in which doctors prescribed treatments that locals considered unnecessary, leading to darkly comic exchanges. For example, one anecdote ridiculed a Spanish physician who arrived with arrogant attitude, dismissing the remedies and treatments of the Indies. This individual consumed all types of food indiscriminately, guided solely by theoretical knowledge, without considering the opinions of the local population. He derided their advice, particularly that provided by the indigenous people. Then, as a result of eating bad fruit and making a poor diagnosis, he died.^42^

In *Secretos*, Benavides also spoke highly of several indigenous healers who, based on their experience and knowledge of local plants, were remarkably effective in treating endemic diseases in the Indies.^43^ He mentioned a case where an Indian doctor successfully treated Viceroy Don Antonio de Mendoza, emphasizing the effectiveness of local healers' remedies over Spanish medicine.

An Indian from the Marquezado de Cuernavaca possesses this cure as a highly guarded secret, and I learned of it from one of his daughters. This Indian was compensated as if he were a very renowned physician…He was the one who successfully treated Viceroy Don Antonio de Mendoza when the European doctors were unable to do so. He transported the viceroy from a location about fourteen leagues away because he was suffering from a *mirarchia*, an ailment that is quite challenging to cure in these parts (Benavides, fol. 61).

In another part of the text, Benavides expressed the lack of trust that the inhabitants of New Spain have in newly arrived European physicians, alluding to the Novohispanic practice of refraining from consulting any doctor for two years after their arrival in New Spain.^44^ Due to their arrogance and ignorance of local plants, physicians often became objects of mockery. Before visiting a recently arrived physician from Spain, some pranksters consumed a large quantity of red prickly pears, resulting in the discoloration of their urine resembling the fruit's color. Upon witnessing this, the physician was so alarmed that he attempted to provide treatment, yet the patient paid no attention, fully aware that this was a jest intended to underscore the physician's lack of knowledge.^45^

In a similar vein, Benavides recounted the exploits of a gentleman residing in the Indies, Antonio de Villafaña, widely renowned for his predilection for practical jokes on recently arrived European physicians, befriending them and inviting them to a meal. Then, he added seasonings or powders to their food that have a laxative effect on his guests, causing them to have to evacuate without having time to reach the restrooms. Nonetheless, the physicians had the opportunity to get their revenge on one occasion when a Dominican monk, aware of the deception, switched his plates with those of Villafaña without him noticing, causing a nearly fatal effect on the trickster gentleman.^46^ Villafaña used these jokes to highlight European doctors’ limited understanding of American diseases and diets, as well as their inability to prescribe effective treatments—even for themselves. According to Pérez Marín, these anecdotes were part of a discursive style used in the early explorations of America (Pérez Marín, 2020, p. 44–45). In any case, these anecdotes expressed distrust and criticism in a straightforward, albeit non-academic manner. Eventually, the lack of trust in traditional medical establishments led a significant portion of the population in New Spain to prefer being attended by barbers, midwives, indigenous healers, and surgeons. Benavides recognized in these anecdotes a collective legacy of evidence pointing to the effectiveness of Indigenous medical traditions—a form of social epistemology, which he also drew upon to defend the value of Indigenous knowledge and articulate his critiques against medical orthodoxy.

## Conclusion

According to Pérez Marín (2020), Benavides should not be regarded as a main precursor of the seventeenth-century Scientific Revolution solely for his reliance on empirical practice (p. 24), since by the mid-sixteenth century medical humanists had already begun to value experience as a source of discovery (Maravall, 1986, p. 460). While this paper does not intend to present Benavides as the “principal precursor” of the Scientific Revolution, nor do we know whether such a figure of “main precursor” can be identified, it is evident that he was among the first surgeons whose practical experience in the New World gave rise to a significant work that challenged scholastic principles. The impact of this work has received little scholarly attention—an issue this paper has aimed to address.

Benavides’ skepticism toward the limited effectiveness of theory-based Renaissance medicine can also be attributed to his frustration with the lack of recognition for his practical expertise by the theoretical medical establishment. Consequently, he seized every opportunity to emphasize the efficacy of surgery over the traditional approaches of academic medicine. This paper highlights two main reasons why Pedro Arias de Benavides' work is particularly relevant. Firstly, given the lack of materials and infrastructure that were readily available in Europe and the impact of humanistic medical thought, he sought to experiment with new therapeutic plants and techniques in the Indies to develop new medical treatment that could be also applied in the Peninsula. Secondly, while Arias de Benavides acknowledged his indebtedness to the medical tradition, he was also convinced that the accomplishments of ancient physicians can be surpassed through experimentation and by acquiring knowledge of the plants and techniques used by indigenous people in the Americas. This supported his keen interest in disseminating and integrating this knowledge into the medical practices of his time.

Importantly, the validation of therapeutic properties that Benavides attributed to indigenous American plants relied on his own experience, despite consistently citing classical authorities whenever possible. In addition, being aware of the hierarchical structure of the medical profession and recognizing that as a surgeon, he had limited possibilities to present his critiques through conceptual discussions, he found in local anecdotes a source of accumulated evidence that substantiated his skepticism about the ineffectiveness of European doctors in the Indies. It can further be argued that, in practical matters, Benavides offered relevant insights in areas where the wisdom of classical authors proved insufficient. As Pérez Marín (2020) correctly points out (p. 23), he criticized those who rely on the testimonies of the authorities when writing, in contrast to his approach which was to observe and experiment with American *materia medica* and indigenous healing techniques. In this manner, Benavides casts doubt on treatments that relied solely on books, thus remaining open to acknowledging the efficacy of numerous remedies employed by Native American Indians, often asserting their therapeutic superiority over classical authorities.

In conclusion, the work of Benavides contributed to the Western assimilation of therapeutic and culinary practices of Native American cultures, which in turn, improved the medical treatment of Spanish and European medicine more generally. *Secretos* vividly illustrates this scientific effort and cultural amalgamation.

## Footnotes

1 *Secrets of surgery, especially of the diseases of morbo gallico and lamparones and mirrachia, and likewise how the Indians cure sores and wounds and other passions in the Indies, very useful and profitable for Spain, and many other Secrets of surgery not yet written.*

2 *Secretos* is part of the medical works published in the second half of the sixteenth century, such as *Libellus de Medicinalibus Indorum Herbis* (1552) by the Nahuatl physician Martín de la Cruz; *Historia medicinal de las cosas que se traen de nuestras Indias Occidentales* (1565) by Nicolás Monardes; *Opera medicinalia* (1570) by Francisco Bravo; *Summa y recopilación de Cirugía* (1578) by Alonso de Hinojoso; and *Quatro libros de la naturaleza y virtudes de las plantas y animales* by Francisco Hernández, posthumously published in 1615, but written during the stay of the author in Mexico from 1571 to 1577.

3 *Secretos* should be seen also as indicative of a more general and growing interest in the sixteenth century in making known in Europe the history and cultural consequences of the encounter between Spain and America, as interpreted by Spanish historians. Some of these works include *Historia general de las Indias* (1555) by Francisco López de Gómara.

4 A general account of the American *materia medica* described by Benavides in *Secretos* lies beyond the scope of this paper. For a examination of this subject, see Valverde et al. (1986). For a broader treatment of *materia medica* in the context of New Spain, see De Vos (2021).

5 The effectiveness of indigenous physicians and their herbal medicine was acknowledged by the Spaniards. Hernández Saenz and Foster note that there was an intention to recognize the medical virtues of American botany, but there was also concern about the magical rituals that accompanied the healing of an illness. In any case, hispanic physicians aimed to weigh and understand the effects of American medicinal substances based on their humoral effects (Hernández Saenz & Foster, 2001, p. 21–22).

6 Comparative studies on the differences between Spanish medicine and the therapeutic knowledge of Native Americans in New Spain include: Aguirre Beltrán (1963), Viesca Treviño (1997), and Viesca Treviño, Aranda & Ramos (1999).

7 Hernández Saenz and Foster (2001, p. 25) note that medical education with a predominantly Spanish curriculum began in 1536 at the Franciscan Colegio de Santa Cruz in Tlaltelolco. In this institution, members of the Tlaxcaltec and Aztec nobility were instructed in Spanish, Latin, theology, rhetoric, philosophy and music.

8 For further information on the developments of Spanish medicine in New Spain, see Viesca Treviño and Aceves-Pastrana (2011), Rodríguez-Sala (2011), González González (1992), and Somolinos Palencia (1989).

9 During the mid-sixteenth century in Spain, there was a distinction within the surgical profession. On the one hand, there were those who held university degrees and were proficient in Latin, referred to as *cirujanos latinos*. On the other hand, there were those who gained their knowledge through hands-on experience working alongside physicians, known as *cirujanos romancistas*. Benavides´ familiarity with foundational medical texts and apparent fluency in Latin suggest that he belonged to the former group (Hernández Saenz & Foster, 2001, p.30). It is worth noting that the literature on surgery was disseminated not only in Latin but also in vernacular languages. This practice aimed to legitimize surgical practice in the eyes of both contemporary and historical medical authorities (Siraisi, 1990, p. 167). There were also distinctions among surgeons themselves, i.e. literate practitioners sought regulations to differentiate themselves from barbers and other non-university-trained medical personnel (Siraisi, 1990, p. 177).

10 On the reception and new interpretation of Galenic medical thought in Spain and New Spain at the end of the Middle Ages and the Renaissance, see Romero y Huesca et al., (2017).

11 Nicolás Monardes (1493–1588) was a prominent and well-known medical figure in the late sixteenth century. Though he never traveled to the Indies, he criticized the lack of experimentation and the disregard for New World *materia medica* (Galeote, 2013, p. 49). He recognized that different regions produced different remedies and emphasized the value of foreign substances (Monardes, 1574, Book 1, fol. 2). Like Benavides, he advocated for a deeper understanding of American botanical knowledge for medical use (Galeote, 2013, pp. 51–52).

12 Francisco Hernández (1516–1587) was best known for his inventory of New World *materia medica*, completed in 1571, following a 1570 expedition through key regions of New Spain, including Oaxaca, Guerrero, Michoacán, central Mexico, and Pánuco (Boumediene, 2020). The key difference between Hernández and Benavides lies in their methods: while Hernández sought to integrate American discoveries into a system grounded in classical thought, Benavides used classical theory only as a starting point, allowing practice to move beyond its boundaries (Viesca Treviño, 1988; Vilchis, 2015).

13 Currently, it is known that syphilis is caused by *Treponema pallidum*, a sexually transmitted bacteria with limited metabolic capacity. Syphilis progresses through five stages. In its early stage, syphilis is recognized by causing lesions on the genitalia. In its late stage, syphilis can cause neurological and cardiological infections. Without treatment, papules and buboes may develop on the body (Rothschild, 2005, p. 1454–1455; French, 2007, p. 143–147). Furthermore, syphilis is, as Boehrer (1990, p. 200) has pointed out, a social disease, as it is transmitted from person to person; social stratification and the movement of people also have a major impact on its virulence. This is compounded by the moral judgment of the infected portrayed as individuals of low morality.

14 ‘…pensar yo escribir cosas de las escritas hasta aquí sería gran yerro pensar querer yo llegar a ninguna parte de las mínimas habilidades de los antiguos escritores’ (Benavides, fol. 62).

15 Although the origin of syphilis is still debated, recent research suggests that it had already appeared in European antiquity (Ioannou et al., 2018). Before the arrival of Europeans in the Americas, there is no evidence that Indigenous peoples contracted the venereal form of the disease, nor are there reports of widespread epidemics. Both the natural and cultural environments of these communities may have played a key role in preventing this development (Powell & Cook, 2005). This highlights the relevance of archaeological, historical, and anthropological studies on the distinct ways human groups have engaged with their biocultural environment. Undoubtedly, these studies can inform current debates in the philosophy of medicine regarding the importance of considering environmental factors as integral to our understanding of health (see Menatti et al., 2022).

16 Martínez Hernández (2022) notes that the conquest of the American territories led to the implementation of new methods of governance for each group. However, this also resulted in a consciousness of racial belonging that was not always inclusive, as seen in the case of Benavides. This in turn led to the creation of medical institutions that served each group, as in the case of the Indians, where European and American sanitary practices were combined.

17 ‘…Santo Domingo, es una de las primeras islas de las indias, tiene nombre de la Isla Española, es tierra muy enferma, en ella ay grandísima cantidad de bubas, la causa dello es que los que nacen en aquella tierra dan a criar a negras, en aquella tierra no he visto a ninguna española que crie a sus hijos, previénense que paran de una negra que tenga la mejor leche que ellos…y luego que paren entregan la criatura a la negra…y la negra siempre tiene el cuidado de criarla, y aunque sea grande siempre la acuesta consigo, y las criaturas comen de los manjares que comen las negras y sus hijos, que son muy malas comidas…se le engendran los humores, y también las mismas negras y sus hijos, todos están llenos de bubas, de lo cual es razón evidente que las han de tener los que tratan y comunican con ellas’ (Benavides, fol.9).

18 ‘Estas vienen por la mayor parte por tener acceso con mujeres no limpias, hay más contagio en tiempo caliente que en frío’ (Benavides, fol. 72).

19 ‘Hay más contagio si se da en tiempo caliente que no en frío, a causa de la relajación de los poros, y la calor grande y el sudor que se recibe en el acto…también esta enfermedad se pega de beber por una vasija que haya bebido el que tuviese la dicha enfermedad, o dormir con quien la tuviere, y así mismo digo que fuera de todas las causas dichas, puede tener la enfermedad cualquiera persona sin contagiosidad ninguna, sino por corrupción de humores que se le corrompa y derrame por el cuerpo’ (Benavides, fol. 72).

20 ‘Formase esta enfermedad a causa de la melancolía, que es el más rebelde y más terrestre humor de todos, verdad es, que hay muchas complexiones de humores diferentes con mixtura unos de otros, de la tierra y el tiempo en que está, a todas se ha de tener intención en la cura. Los que curan en tiempo algo frío, tienen las carnes más tiesas más clausuras en los poros, la naturaleza más fuerte, los espíritus vitales más recogidos, y el calor natural metido en el centro y más fortificado y muy bastante para expeler el humor por las partes inferiores’ (Benavides, fol. 82).

21 For Benavides, the hot land and food was responsible for the majority of diseases and deaths among young people in the Indies: ‘los jóvenes que van de acá corren gran riesgo a causa del gran calor que consume el húmedo radical’ (Benavides, fol.10). In contrast, this territory is ‘muy buena para los viejos que pasan allá… que sobreviven muy sanos’ (Benavides, fol. 10).

22 Fresquet (1993, p. 127) argued that most Spanish physicians' reports of syphilis failed to recognize the first stages of the disease, specifically the formation of chancres. However, they were very effective at describing its later manifestations.

23 ‘Las señales son a los principios algún mal de verga, algunos encordios, dolores en junturas, caimiento de las cejas y pestañas, a manera de alopecia, algunas llagas en la campanilla, de mala digestión, algunos dolores de cabeza sin fiebre, algunos nudos que empiezan a salir en la cabeza, aunque pequeños, con muy mala color en la cara, y flojedad…Cuando esta enfermedad cae en hombre melancólico y algo en la edad téngola por muy dificultosa cura, en hombres mal reglados y bebedores de vino es labor en balde…Las mujeres por la mayor parte se curan bien de esta enfermedad…porque acontece por razón de la enfermedad habérseles alzado su regla, y como dicho tengo, con la cura vuélveles a bajar y naturaleza por allí expele y lanza cualquier cosa que quede’ (Benavides, fol. 71. 73).

24 ‘Las causas de esta enfermedad por la mayor parte “per paulatim congestionen” y hallarse más presto, o en la mayor parte en hombres blancos, de color flemáticos, es un humor muy rebelde de curar’ (Benavides, fol. 83).

25 ‘[Las] causas de la enfermedad son humores gruesos que se sientan sobre el cráneo, o en alguna espinilla. Véase el humor grueso y con todo esto hace el asiento y corrupción en los huesos y los corroe y corrompe y hace gibosos…La intención que tengo en esta cura es esta: Si veo que tienen alguna inflamación y que no están aún sanos, procuro hacer evacuación con sangrías de venas en todo el cuerpo…luego procuro digerir con jarabe de borrajas…y estando digesto el humor, se conocerá por la orina…tome luego unas píldoras…colchicas y sumarias…si los nudos viere el cirujano se van deshaciendo, deben tomar otra vez a purgarle y encima del nudo ponerle un parche de diaquilón amoniacado, de meliloto…cuezan luego en una olla grande…una onza de zarzaparrilla lavada y de ella le den a beber lo que quisiese, y muelan cuatro o seis onzas de zarzaparrilla y háganla harina y cada mañana en una copa de agua cocida echen una cucharada pequeña del polvo…y bébanlo una vez en la mañana y una vez en la noche’ (Benavides, fol. 76).

26 ‘Anse guardar todas las cosas saladas y agrias y gruesas que engendran malos humores, por una distancia de más de dos meses, para que en ese tiempo se engendre buen humor en el cuerpo humano…no ha de comer tocino ni pescado, fresco ni salado, ni vinagre, ni cosa áceda, ni carne de vaca, ni frutas…no beber vino en todo ese tiempo, y también se ha de guardar de comer legumbres, así como habas, lentejas y otras cosas de esta manera…ni coma ningún género de verduras salvo borrajas, hierbabuena, perejil, chicoria y escarolas’ (Benavides, fol. 74).

27 ‘Este humor se digiere…con una poción de confecciones de amech y letuario indio…encima un poco de ungüento amarillo, o blanco, litargino…se deben guardar el vino…en cuanto montan esta cura conozco cuando quedan buenos, que salen muy gordos, y que las llagas se les cierran sin medicinas gruesas’ (Benavides, fol. 86).

28 ‘Cuando están los enfermos en esta disposición se les debe alargar la comida, dándoles caldos de aves, o de carnero…y sus huesos frescos a todo tiempo…y téngase en cuenta la orina…porque es maravillosa señal que el hígado se limpia’ (Benavides, fol. 81).

29 ‘Yo si curase semejantes enfermedades…procuraría la evacuación universal, y al principio sus sangrías’ (Benavides, fol. 86).

30 ‘…tienen unos guaymaros, que es unos ungüentos que hacen de una yerba que acá llaman berraza, sácanla el zumo y muelen otra yerba que acá llaman cebadilla y cuélanlo todo y con aceite y con cera al fuego lo cuajan…La cebadilla es muy venenosa y es cáustico porque con ella se curan las llagas a los caballos y les matan los gusanos que se crían en las mataduras, esta es cura de negros e indios’ (Benavides, fol. 11–12).

31 ‘Los que no tienen llagas, sino dolores y bubas curanse con una hierba…que se llama hierba hedionda, cortaban aquellas varas que son pequeñas y hacían un hoyo en el suelo y ponían allí un poco de lumbre…y sentaban al que querían curar de piernas abiertas…y luego empezaban a estornudar, y lanzar por la boca y narices, muchos humores malos’ (Benavides, fol. 12).

32 The use of wood from this tree became known, and somehow popular, in Europe through the chronicles of Fernández de Oviedo and Ulrich von Hutten's *De guaiaci medicina et morbo gallico*. Its popularity led the Fulger bankers to obtain a monopoly on its trade (Parascandola, 2009, p. 15–16).

33 ‘El palo de las Indias se tuvo en estas partes por medicina muy loable para enfermedades de bubas y otras pasiones flemáticas y de junturas, y dicen y afirman, ser medicina carminativa y que se curan con ella radicalmente…paréceme que si llevasen por su estilo sin faltar cosa alguna, una razonable enfermedad se curaría sin gota alguna del palo, con solo la dieta y las purgas que le manda dar al principio, antes de entrar a beber el agua y otra vez al medio de la cura y otra salir de ella…creo a mi parecer cura más la dieta que el Palo de las Indias’ (Benavides, fol.18-19).

34 ‘Otros tienen otra manera de curarse y que toman zarzaparrilla y échanla en remojo cuando está fresca en unas piedras que allá llaman metates…como sale el mucílago del malvavisco…sin más purgar ni otra cosa, le dan cada mañana al enfermo que se cura, cantidad de media azumbre…Esta toman tres días sucesivamente sin comer ni beber cosa alguna salvo agua simple de zarzaparrilla’ (Benavides, fol.17).

35 ‘Nunca cesan de sudar y pasados estos días hacen gran cantidad de cámaras y quedan buenos’ (Benavides, fol.17).

36 ‘Otros, la tuestan [la zarzaparrilla] y hacen harina, que se hace como de cebada y tiene buen sabor y cada vez que la beben la echan en agua o con el vino tanta cantidad como quepa en una cascara de nuez y esta tómanla sin dieta…dos o tres meses y al cabo de dicho tiempo quedan bien curados y de esa suerte yo la he dado a muchas personas y me he hallado bien con el suceso de la cura’ (Benavides, fol.17–18).

37 ‘Débase digerir este humor [melancólico]…con jarabe de palomina y de borrajas, de cada uno una onza o más, según la disposición del enfermo y la calidad del humor…estando digesto el humor se purgue con píldoras sumarias…y si hubiese necesidad de purga no se dé en conjunción ni oposición de la luna, ni día crítico…En todo el tiempo que se curan les doy agua cocida de anís o hinojo, y si el enfermo tuviese posibilidad agua de zarzaparrilla…tomen tres cuartas de azogue, pesadas de marco y no medícales, échenlo en un almirez y allí lo amasen con triaca’ (Benavides, fol. 75).

38 ‘No creo que haya árbol en el mundo con tantas virtudes, ni propiedades, ni que de tantas cosas sirva…este es el principal tesoro de Indias’ (Benavides, fol. 40).

39 It is very likely, as Fresquet remarks, that Benavides had learned about the therapeutic properties of maguey through the chronicles of López de Gómara and Pedro Cieza de León, before personally experiencing them (Fresquet, 1993, p.105).

40 ‘Las pencas que cortaron, sécanlas, enderézanlas y hacen tablas para techar sus casas…De las puntas hacían agujas para coser’ (Benavides, fol. 42–44).

41 Currently, agave remains culturally significant in the regions where it is cultivated, valued not only for products like aguamiel, mezcal and pulque but also as a vital part of Indigenous biocultural heritage, especially among the Nahua (Sánchez Jiménez, 2025, pp.1983–1984). Furthermore the relevance of maguey, and the Indigenous knowledge associated with this plant, are important topics in ethnobotanical discussions. For instance, aguamiel is valued for its medicinal properties, used to treat anemia, diabetes, intestinal parasites, and coughs, and is also believed to aid digestion, circulation, and appetite. When applied topically, it also serves as a healing salve (Vázquez García et al., 2016, p. 77).

42 Un médico muy famoso, y él iba tan soberbio y satisfecho de su habilidad, que antes que llegase a México empezó a amenazar a médicos y cirujanos…Como aportó a las Indias, él comió muchas frutas malas de la tierra, y diérole unas cámaras coléricas y el fiándose en su habilidad, como dicho tengo, aunque hubo algunos médicos que le certificaron los sucesos de aquella enfermedad en aquella tierra, y él les respondia, váyanse vuestras mercedes con Dios, y así metió en una tina de agua fría hasta el estómago, y como estaba tan descuidado de ahí salió muerto’ (Benavides, fol.53).

43 For Viesca Treviño (2001), the *curanderos* emerged in the sixteenth century as a result of the conquest and the assimilation of Spanish folk medical knowledge with diverse knowledge from indigenous peoples. The Spaniards labeled all the variants of indigenous medical specialists (Nahuas, Mayas, Huastecs, and Tzeltals, among others), as *curanderos*, overshadowing the cultural and linguistic differences between them.

44 ‘…en las Indias tienen por estilo no curarse con médico alguno, hasta que haya pasado dos años por ellos…que por la mayor parte son mozos [que] hagan experiencia de las cosas tocantes a las medicinas en otros, y no en ellos’ (Benavides, fol. 46).

45 ‘Con las tunas coloradas se han hecho muchas burlas a médicos recién idos de la siguiente manera. Come uno muchas tunas, y orina en un orinal, y llévale la orina, y es como sangre viva, y el médico hace muchos remedios refrigerativos y algunas lobotomías, y él tiene la orina, o cuya es, dice muy disimuladamente dejémoslo por hoy y mañana, que soy muy enemigo de medicinas, y si fuese cosa delante yo llamaré a vuestra merced, y otro día adelante viene el médico a visitar, y dice, señor sin hacerse nada se me ha quitado, y el médico atemorizado y espantado’ (Benavides, fol.46).

46 ‘Un caballero hay en las Indias que se llama Antonio de Villafaña, que es muy donoso, y hace muchas burlas a médicos nuevos que van desta tierra que tiene por estilo de convidarlos a comer, y luego se hace amigo de él, y está siempre prevenido de unos polvos de habas que hace echar en el potaje convidado, que no ha bien acabado de comer, cuando tiene necesidad de desencintar las calzas, porque la prisa es tanta, que no tiene remedio…A un fraile domínico convidó y este mismo caballero y el fraile estaba avisado de la burla…le sirvieron un potaje, y como el fraile entendió que ahí venía la burla, muy disimuladamente pasó su servicio al caballero…fue tanta la burla que llegó Villafaña a punto de muerte, y de esta manera se vengaron los médicos’ (Benavides, fol.46–48).
